# *Talaromyces* sp. Ethyl Acetate Crude Extract as Potential Mosquitocide to Control *Culex pipiens quinquefasciatus*

**DOI:** 10.3390/molecules28186642

**Published:** 2023-09-15

**Authors:** Junhui Chen, Zhiyong Xu, Yangqing Liu, Feiying Yang, Limei Guan, Jian Yang, Jianghuai Li, Guodong Niu, Jun Li, Liang Jin

**Affiliations:** 1 Institute of Biological Resources, Jiangxi Academy of Sciences, Nanchang 330929, China; allenchen0426@gmail.com (J.C.); 18059141865@163.com (F.Y.); glmnh@126.com (L.G.); jemappelleyangjian@zju.edu.cn (J.Y.); jeremy_leakey@sina.com (J.L.); 2 Institute of Applied Chemistry, Jiangxi Academy of Sciences, Nanchang 330929, China; zhiyongxuconfident@hotmail.com; 3 Nanchang Center for Disease Control and Prevention, Nanchang 330100, China; nccdclyq@163.com; 4 Department of Biological Sciences, Florida International University, Miami, FL 33199, USA; gniu@fiu.edu (G.N.); lij@fiu.edu (J.L.)

**Keywords:** fungal metabolites, vector control, vermistatin, mosquitocides

## Abstract

Vector control is considered an effective approach to controlling diseases spread by mosquito bites. Entomopathogenic fungi are widely used in agriculture to control insect pests, and fungal metabolites can potentially be developed as effective mosquitocides. In this study, a high-throughput screening method was used to search for potential mosquitocides in the Global Fungal Extract Library (GFEL). We tested the larvicidal activity of 264 fungal ethyl acetate crude extracts against *Culex pipiens quinquefasciatus*. Nine fungal extracts caused moderate to high mortality rates (>50%), with two fungal extracts (58A7 and 101H12) causing a 100% mortality rate. The lethal concentrations for 50% of the population (LC_50_) were 44.27 mg/L and 31.90 mg/L, respectively. Fraction 14 had a high mortality rate, with an LC_50_ value of 12.13 mg/L, and was isolated from 58A7 (Fractions 1–11) and 101H12 (Fractions 12–15). Further analyses showed that Fraction 14 was made up of vermistatin and dihydrovermistatin. In a *Cx. p. quinquefasciatus* larvicidal bioassay, vermistatin (LC_50_ = 28.13 mg/L) was more toxic than dihydrovermistatin (LC_50_ = 83.87 mg/L). Our findings suggested that the active fungal extract 101H12 from *Talaromyces* sp. and its compound vermistatin could be developed as mosquitocides.

## 1. Introduction

Mosquitoes are important vectors for transmitting a wide range of serious human and animal diseases (e.g., malaria, dengue), posing a threat to public health. Malaria killed more than 400,000 people in 2019 [1]. *Culex pipiens quinquefasciatus,* known as the ‘southern house mosquito’, is distributed throughout southern China. *Cx. p. quinquefasciatus* has been identified as a vector for the transmission of St. Louis encephalitis and the West Nile virus in North America [2,3], as well as Japanese encephalitis and the Zika virus in China [4]. 

Due to the lack of vaccines, vector control has been widely used in the prevention of vector-borne diseases and has proven to be a successful strategy in reducing death cases globally [5]. In Africa, insecticide-treated nets are the most important contributor to indoor residual spraying and artemisinin-based combination therapy in reducing clinical malaria cases [6]. However, the extensive use of chemicals has resulted in environmental pollution and an increase in resistance. In Réunion Island, *Cx. p. quinquefasciatus* has developed a 233-fold resistance against permethrin, deltamethrin, temephos, chlorpyrifos, malathion, and dieldrin. In addition, the vector population could retain high dieldrin-resistant Rdl^R^ frequencies for decades [7], implying that new vector control strategies should be implemented. 

Natural biological resources are considered efficient methods to overcome insecticide resistance and environmental pollution, so they are frequently used for pesticide discovery. Fungi have numerous species [8,9], diverse secondary metabolites [10] and large-scale fermentation [11]. Many fungal genera, including *Lagenidium*, *Coelomomyces*, *Conidiobolus*, *Entomophthora*, *Culicinomyces*, *Erynia*, *Beauveria*, and *Metarhizium* are toxic to mosquitoes like *Anopheles*, *Culex*, and *Aedes* [12]. The synthesis and secretion of active secondary metabolites such as destruxins, efrapeptins, beauvericin, and beauveriolides are toxic to mosquitoes [13]. 

The Global Fungal Extract Library (GFEL) contains over 10,000 fungal isolates including *Penicillium* spp., *Aspergillus* spp., *Fusarium* spp., *Podospora* spp., *Mucor* spp., *Cladosporium* spp., and *Stoloniferum* spp. [14]. Among these fungal metabolites, asperaculane B, extracted from *Aspergillus aculeatus*, has been widely used in malaria control as it inhibits malaria infection and transmission [15]. Jin et al. [16] discovered six fungal extracts with high toxicity against the *An. gambiae* Sua5B cell line after screening 192 fungal extracts for effective *An. gambiae* insecticide discovery. Among these extracts, *Penicillium toxicarium* 76F6 extract showed high larvicidal and adulticidal activity against *An. gambiae*. The GFEL is an essential resource for novel insecticide discovery in the control of mosquitoes.

To discover new insecticides against *Cx. p. quinquefasciatus* with a friendly profile for humans and the environment, we used conventional bioassays on mosquito larvae to screen 264 candidate fungal extracts for the most effective fungal metabolites. Following the discovery of these candidate metabolites, we then isolated the active compounds and validated their larvicidal activities. 

## 2. Results

**Figure 1:**
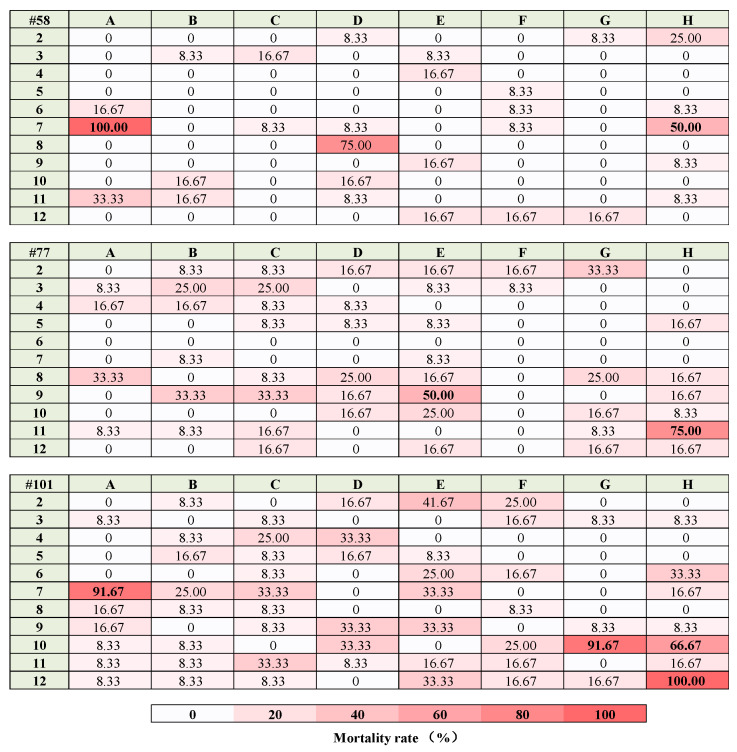
Toxicity of 264 fungal crude extracts in ethyl acetate against fourth-instar larvae of *Culex pipiens quinquefasciatus* at 100 mg/L. Numbers indicate the percentage of mortality rate. Lighter red colors indicate a lower mortality rate, and darker red colors indicate a higher mortality rate.

**Figure 2:**
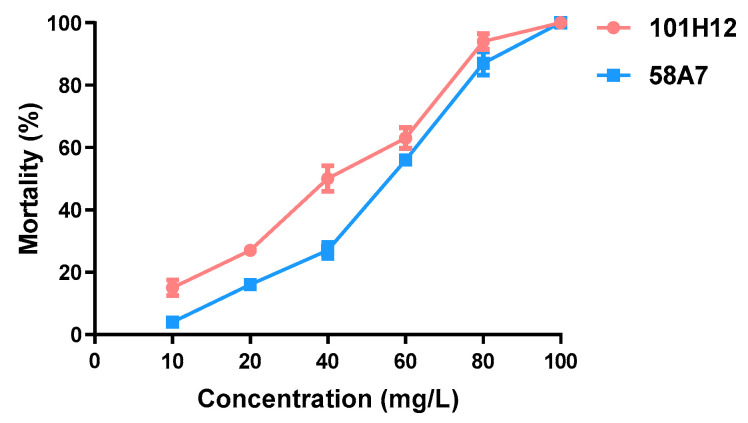
Larvicidal bioassay of 101H12 and 58A7 against fourth-instar larvae of *Culex pipiens quinquefasciatus*. Batches of 25 fourth-instar larvae were tested and four replicates were conducted. Mortality was recorded after 24 h. Standard probit analysis was conducted.

In the 264 tested ethyl acetate crude extracts, nine fungal extracts caused a mortality rate of more than 50%, and two fungal extracts caused a 100% mortality rate at 100 mg/L (Figure 1). Further larvicidal bioassays of 101H12 and 58A7 confirmed that both mortality rates reached 100% at 100 mg/L (Figure 2). The LC_50_ value of 101H12 was 31.90 mg/L with 95% CL 26.957~37.14 mg/L and the value of 58A7 was 44.27 mg/L with 95% CL 38.39~50.67 mg/L (Table 1).

**Figure 3:**
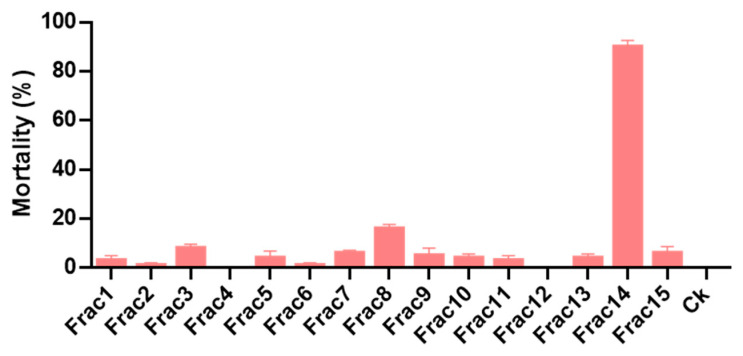
Toxicity of 15 fractions of 101H12 and 58A7 against fourth-instar larvae of *Culex pipiens quinquefasciatus* at a concentration of 20 mg/L.

A total of 15 fractions were separated, among which 58A7 contained eleven fractions (Fractions 1–11) and 101H12 contained four fractions (Fractions 12–15). Toxicity results showed that Fraction 14 caused a 90% mortality rate at 20 mg/L and a 100% mortality rate at 30 mg/L (Figure 3). The LC_50_ of Fraction 14 was 12.13 mg/L with 95% CL 10.89~13.40 mg/L. 

**Figure 4:**
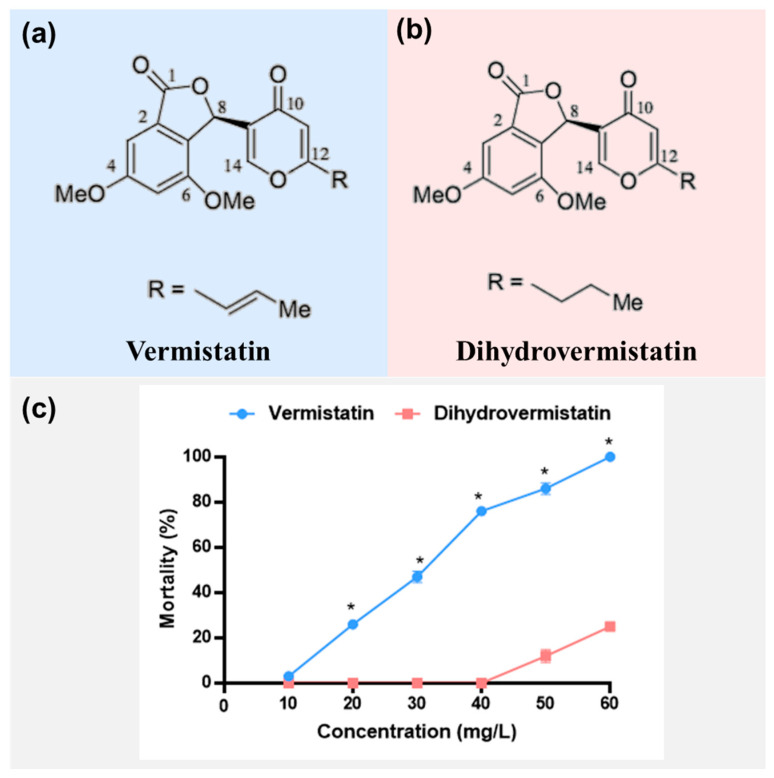
Chemical structures of vermistatin (**a**) and dihydrovermistatin (**b**) and their larvicidal bioassay against fourth-instar larvae of *Culex pipiens quinquefasciatus* (**c**). “*” indicates significant differences at the same concentrations (*p* < 0.05).

Further separation of Fraction 14 found two subfractions. The identification by hydrogen spectrum and carbon spectrum showed that Fraction 14.1 was vermistatin and Fraction 14.2 was dihydrovermistatin (Figure 4a,b). Larvicidal bioassay showed that vermistatin was much more toxic than dihydrovermistatin (*p* < 0.05) (Figure 4c). The LC_50_ of vermistatin was 28.13 mg/L with 95% CL 26.33~29.91 mg/L and that of dihydrovermistatin was 83.87 mg/L with 95% CL 71.66~118.77 mg/L.

## 3. Discussion

The screening of active compounds from fungal extracts has received more and more interest and the GFEL has proven to be a successful resource. Niu et al. [14] screened 460 fungal extracts from the GFEL, found that four fungal extracts showed more than 90% inhibition activity in preventing fibrinogen-related protein 1 from binding to *Plasmodium falciparum* lysates, and eventually found that *Penicillium thomii* extract can block *Plasmodium falciparum* to transmit to *An. gambiae*. Jin et al. [16] screened 192 fungal extracts from the GFEL, established an MTT-based cytotoxicity screening approach, and found that *Penicillium toxicarium* extract exhibit high toxicity to mosquito larvae and adults of *An. gambiae*. Similarly, *Aspergillus aculeatus* extract completely inhibited *Plasmodium falciparum* transmission to *An. gambiae*. Asperaculane B was successfully purified and identified, with a half maximal inhibitory concentration (IC_50_) of 7.89 µM for *Penicillium falciparum* transmission and an IC_50_ of 3 µM for the development of asexual *Penicillium falciparum* [15]. In addition, sterigmatocystin and pulixin, isolated from *Penicillium janthinellum* extract and *Purpureocillium lilacinum* extract, respectively, limited *Plasmodium falciparum* proliferation, transmission, or infection [14,15]. In this study, we ultimately discovered that nine candidates were able to kill more than half of the larval mosquitoes at a concentration of 100 mg/L. Only 101H12 (*Talaromyces* sp.) and 58A7, causing 100% mortality, were chosen for further mosquito larvicidal bioassays. The other seven fungal extracts showed medium-to-high toxic activity, which also deserves a comprehensive study. Future investigations are required to determine the larvicidal effects of the remaining seven fungal extracts and identify their active compounds, assessing their potential use in mosquito control. 

*Talaromyces* contains over 200 secondary metabolites, including esters, terpenes, steroids, alkaloids, polyketides, and anthraquinones [17]. Some compounds have biological activities, such as anti-inflammatory, bacteriostatic, and antitumor activities [18]. The compounds of *Talaromyces* sp. strain LF458 display potent antibacterial activities against *Staphylococcus* strains and acetylcholinesterase inhibitory activities [19]. 

Fuska et al. [20,21] separated vermistatin from *Penicillium vermiculatum* and elucidated its structure in 1979. Besides *Penicillium*, vermistatin has been found in other taxa such as *Talaromyces thailandensis* [22], *Guignardia* sp. [23], *Eurotium rubrum* [24], and co-culture of *Alternaria alternate* and *Streptomyces exfoliates* [25] and co-culture of *Penicillium* sp. and *Streptomyces fradiae* [26]. In correspondence with our results, vermistatin showed biological activities such as cytotoxic activities against tumor cells [23] and strong inhibitory activity against α-glucosidase [24]. However, vermistatin showed no anti-fungal activities against *Saccharomyces cerevisiae*, *Aspergillus niger*, *Staphylococcus aureus* [23], and *Candida albicans*, but it potentiated the anti-*Candida albicans* activity of miconazole [27]. 

Vermistatin is often extracted together with derivatives. Dihydrovermistatin, one of its derivatives, is often extracted together with vermistatin from *Alternaria alternate* [25], *Penicillium rubrum* [28], and *Phoma* sp. [29]. In addition to dihydrovermistatin, other derivatives such as 6-demethylvermistatin [30], methoxyvermistatin, and hydroxyvermistatin [23] have been separated. In our results, dihydrovermistatin exhibited low biological activity against *Cx. p. quinquefasciatus*. Similarly, dihydrovermistatin exhibited no activity against leukemia cell lines [28]. The probable reason is that the structure of the propenyl tail in dihydrovermistatin causes biological inactivity [31]. In comparison with the propenyl tail, the methoxyl group at the γ-pyrone ring exhibited a higher cytotoxic activity in methoxyvermistatin than in vermistatin against the KB tumor cell line, with IC_50_ values of 20.0 and 90.2 mg/mL, respectively [23]. 

In our study, a mixture of vermistatin and dihydrovermistatin (Fraction 14, LC_50_ = 12.13 mg/L) showed higher larvicidal activity than the crude fungal extract (LC_50_ = 31.90 mg/L) and the single compounds vermistatin (LC_50_ = 28.13 mg/L) and dihydrovermistatin (LC_50_ = 83.87 mg/L), indicating that the mixture may be more suitable for further mosquitocide development. In addition, the mechanism behind this deserves further study. 

The efficacy of *Talaromyces* sp. extract or vermistatin needs to be improved for their further application. Synergists such as piperonyl butoxide, a well-known cytochrome P450 inhibitor, S,S,S-tributylphosphorotrithioate, an esterase-specific inhibitor, and diethyl maleate, a glutathione S-transferases inhibitor have been proved to be effective synergists in mosquito control [32,33]. Nanoencapsulation has been employed to protect active compounds from external factors and to maximize their biological activity. For example, nanoliposomes of carvacrol showed a better larvicidal efficacy against *An. stephensi* and *Cx. quinquefasciatus* [34]. Hence, a broad source of active compounds can be synergized and nanoencapsulated for potential mosquitocides.

## 4. Materials and Methods

### 4.1. Rearing Mosquitos

*Cx. p. quinquefasciatus* was obtained from the Nanchang Center for Disease Control and Prevention. Mosquitos had originally been collected in Nanchang, South China, and have been reared for more than 10 years. An artificial diet of yeast:lactose albumin (1:1) was used to rear *Cx. p. quinquefasciatus* larvae. Mosquitoes were kept in the insect room at 26 °C, 65% relative humidity (RH), and a photoperiod of 12 h light followed by 12 h dark. Larvae were fed with 0.1 mg ground fish food. Adults were fed with 8% sucrose and mouse blood for egg production. 

### 4.2. Fungal Culture and Metabolite Extraction 

Ethyl acetate crude extracts of fungi were acquired from the Global Fungal Extract Library (GFEL, Aulandin, Jiashan, China) [14]. The detailed fermentation process was described previously [15]. Five hundred grams of cereal (General Mills, Minneapolis, MN, USA) was sterilized at 121 °C for 20 min. When the cereal was dried, it was mixed with 1 L 0.3% sterile sucrose solution supplemented with 50 mg/L chloramphenicol (Life Tech, Grand Island, NY, USA). Fungal cultures were kept in a mushroom bag at 27 °C for 4 weeks. One liter of ethyl acetate was added to the culture twice to extract fungal metabolites. The ethyl acetate solution was filtered using a Büchne Flask with two layers of cheesecloth and the solid residue was discarded. A rotary evaporator (Heidolph, Elk Grove Village, IL, USA) was used to eliminate the resultant ethyl acetate supernatant and to acquire crude gums. 

### 4.3. Isolation and Identification of Compounds

The ethyl acetate extracts were subjected to vacuum liquid chromatography (VLC) on silica gel using a step gradient of petroleum ether/ethyl acetate (9:1, 8:2, 7:3, 6:4, 5:5). Fractions 1–11 belong to extract 58A7 (5 g) and fractions 12–15 belong to extract 101H12. Subsequently, vermistatin (18.7 mg, t_R_ 32.4 min) and dihyrovermistatin (6.8 mg, t_R_ 37.6 min) were obtained from Fraction 14 using a preparative RP-C18 HPLC (CH_3_CN–H_2_O, 35:65). Their structures were identified by hydrogen–carbon spectroscopy and literature comparison [35]. TLC (petroleum ether/ethyl acetate (5:5, *v*/*v*)): Rf (vermistatin) = 0.58, Rf (dihyrovermistatin) = 0.62. Structures of the compounds were identified based on NMR spectroscopic methods, mass spectrometry, as well as by comparison with the literature data (See the Figures S1–S4 in Supplementary Materials).

Vermistatin: white crystal; ^1^H NMR (400 MHz, CDCl_3_) *δ*_H_ 7.36 (1H, s, H-14), 7.19 (1H, s, H-3), 6.91 (1H, s, H-5), 6.61 (1H, s, H-8), 6.54 (1H, m, H-16), 6.10 (1H, s, H-11), 6.00 (1H, d, *J* = 15.6 Hz, H-15), 3.81 (3H, s, 4-OCH_3_), 3.72 (3H, s, 6-OCH_3_) 1.86 (3H, s, H-17); ^13^C-NMR (100 MHz, CDCl_3_) *δ*_C_ 177.4 (C-10), 170.2 (C-12), 163.2 (C-14), 162.3 (C-1), 155.0 (C-4), 154.0 (C-6), 136.1 (C-16), 129.5 (C-2), 127.9 (C-9), 123.6 (C-15), 123.3 (C-7), 113.0 (C-3), 105.3 (C-5), 99.2 (C-11), 73.7 (C-8), 56.1 (4-OCH_3_), 55.9 (6-OCH_3_), 18.7 (C-17).
Dihyrovermistatin: white solid; ^1^H NMR (400 MHz, CDCl_3_) *δ*_H_ 7.36 (1H, s, H-14), 6.91 (1H, s, H-3), 6.61 (1H, s, H-5), 6.40 (1H, s, H-8), 6.13 (1H, s, H-11), 3.81 (3H, s, 4-OCH_3_), 3.72 (3H, s, 6-OCH_3_), 2.41 (2H, m, H-15), 1.60 (2H, m, H-16), 0.91 (3H, t, *J* = 7.4 Hz, H-17); ^13^C-NMR (100 MHz, CDCl_3_) *δ*_C_ 177.1 (C-12), 170.2 (C-10), 169.5 (C-14), 163.2 (C-1), 155.0 (C-4), 154.5 (C-6), 129.5 (C-2), 127.9 (C-9), 123.6 (C-7), 114.5 (C-12), 105.3 (C-3), 99.2 (C-5), 73.7 (C-8), 56.1 (4-OCH3), 55.9 (6-OCH3), 35.4 (C-15), 20.1 (C-16), 13.5 (C-17).

### 4.4. Larvicidal Bioassay

The mosquito larval bioassay was conducted based on the requirements of the WHO with slight modifications [36]. For the initial screening, a 24-well plate was used, and each well contained 2 mL of distilled water. Ethyl acetate crude extracts of fungi were dissolved in dimethyl sulfoxide (DMSO, 99%AR, Hengxing company, Tianjin, China) and were pipetted into the wells to make final concentrations at 100 mg/L. Twelve 4th-instar larvae were transferred into the wells and mortality was recorded after 24 h. 

The efficacy of the screened fungal extracts, namely 101H12 and 58A7, was evaluated across a range of concentrations (10, 20, 40, 60, 80, and 100 mg/L) in 100 mL of distilled water using glass beakers. Similarly, the isolated fractions 1–15 were initially tested at a concentration of 20 mg/L, and a series of concentrations (5, 10, 15, 20, and 30 mg/L) of Fraction 14 and of its components vermistatin and dihydrovermistatin (10, 20, 30, 40, 50 and 60 mg/L) were tested. Negative control was 1% DMSO without any fungal extract. Batches of 25 4th-instar larvae were tested and 4 replicates were conducted. Mortality was recorded after 24 h if no reaction was seen when the larvae were touched with a soft plastic dropper. No food was provided during the experiment. Every test was replicated three times on different days.

### 4.5. Identification of Fungal Species

The conserved sequences of the internal transcribed spacer (ITS) region of 5.8S and 28S ribosomal DNA were used to identify individual fungal species. A small amount of mycelium (0.1–1 mg) was taken from fungus 101H12, rinsed in 400 μL sterilized water, and then collected by centrifugation at 15,000× *g* for 2 min. The mycelium was resuspended in 100 μL sterilized water, 1 μL of which was used for PCR. The DNA fragments were amplified using ITS1 (TCC GTAGGT GAA CCT GCG G) and ITS4 (TCC TCC GCT TATTGA TAT GC) primers. The reaction was carried out under the following conditions: 94 °C for 2 min to denature DNA; 35 cycles of 94 °C for 30 s, 55 °C for 30 s, and 72 °C for 1 min; and 72 °C for 5 min to complete the reaction. The amplified PCR product was gel-extracted, purified, and sequenced (Eurofins Genomics, Louisville, KY, USA). The raw sequence data were analyzed, and low-quality sequence ends were removed. Subsequently, the edited sequences were compared with those in the National Center for Biotechnology Information database using BLAST to identify individual fungal species. The fungus was identified as *Talaromyces* sp. and found to be 100% identical to GenBank: ON427045.1. 

### 4.6. Data Analysis

SPSS (version 19.0) was used to analyze the data. Larvicidal bioassay was conducted by standard probit analysis. The lethal concentrations for 50% and 90% mortality (LC_50_ and LC_90_), standard deviation, and 95% confidence intervals (CLs) of the means of LC_50_ and LC_90_ were calculated. GraphPad (version 8.0) was used to generate the figures. 

## 5. Conclusions

In this study, we successfully discovered several potential mosquitocides by a high-throughput screening of fungal extracts from the GFEL. We tested 264 fungal crude extracts in ethyl acetate and found two extracts (101H12 and 58A7) that caused a 100% mortality rate. A total of 15 fractions (Fractions 1–11 from 58A7 and Fractions 12–15 from 101H12) were separated but only Fraction 14 caused a high mortality in the bioassay. Fraction 14 was constituted by vermistatin and dihydrovermistatin. Vermistatin showed strong biological activity in the larvae of *Cx. p. quinquefasciatus*. Our findings indicated that 101H12 *Talaromyces* sp. and its active compound vermistatin are suitable candidates for mosquito control, which will lay the foundations for further development of *Talaromyces* sp. and vermistatin as novel mosquitocides or pesticides.

## Figures and Tables

**Table 1:** Effects of fungal crude extracts and compounds against *Culex pipiens quinquefasciatus*. LC_50_ and LC_90_ values (concentrations that caused mortality in 50% or 90% of a sample population) were determined at 24 h.

Chemical	LC_50_	95% CL	LC_90_	95% CL	Regression Equation	x^2^ Value	*p*
101H2	31.898	26.957~37.137	93.917	76.049~126.177	y = −4.109 + 2.733x	45.713	0.002
58A7	44.269	38.391~50.672	102.008	84.596~133.503	y = −5.819 + 3.535x	53.634	0.000
Fraction 14	12.128	10.894~13.395	22.160	19.432~26.644	y = −5.306 + 4.896x	34.659	0.010
Vermistatin	28.132	26.325~29.912	52.001	47.870~57.588	y = −6.961 + 4.803x	21.772	0.474
Dihydrovermistatin	83.869	71.655~118.768	145.325	106.911~295.192	y = −10.326 + 5.368x	8.960	0.994

## Data Availability

Data are available by request through the authors.

## Supplementary Materials

The following supporting information can be downloaded at https://www.mdpi.com/article/10.3390/molecules28186642/s1: Figure S1: 1H NMR (400 MHz, CDCl3) spectrum of vermistatin. Figure S2: 13C NMR (100 MHz, CDCl3) spectrum of vermistatin. Figure S3: 1H NMR (400 MHz, CDCl3) spectrum of dihyrovermistatin. Figure S4: 13C NMR (100 MHz, CDCl3) spectrum of dihyrovermistatin.

## Sample Availability

Samples of the compounds are available from the authors.
