# Supplementary material for: Talaromyces sp. Ethyl Acetate Crude Extract as Potential Mosquitocide to Control Culex pipiens quinquefasciatus
Source: Molecules. 2023 Sep 15;28(18):6642. doi: 10.3390/molecules28186642 (PMC10534940; doi:10.3390/molecules28186642)
Supplement: Supplementary file 1 [file molecules-28-06642-s001.zip › molecules-2578020-supplementary.pdf]

Talaromyces sp. Ethyl Acetate Crude Extract as Potential Mosquitocide to Control *Culex pipiens quinquefasciatus*

Junhui Chen [1,†], Zhiyong Xu [2,†], Yangqing Liu [3], Feiying Yang [1], Limei Guan [1], Jian Yang [1], Jianghuai Li [1], Guodong Niu [1,4], Jun Li [1,4] and Liang Jin [1,*]

[1] Institute of Biological Resources, Jiangxi Academy of Sciences, Nanchang 330929, China; allenchen0426@gmail.com (J.C.); 18059141865@163.com (F.Y.); glmnh@126.com (L.G.); jemappelleyangjian@zju.edu.cn (J.Y.); jeremy_leakey@sina.com (J.L); gniu@fiu.edu (G.N.); lij@fiu.edu (J.L.)
[2] Institute of Applied Chemistry, Jiangxi Academy of Sciences, Nanchang 330929, China; zhiyongxuconfident@hotmail.com
[3] Nanchang Center for Disease Control and Prevention, Nanchang 330100, China; nccdclyq@163.com
[4] Department of Biological Sciences, Florida International University, Miami, FL 33199, USA
* Correspondence: jinliang079@163.com; Tel.: +86-183-7917-4620
† These authors contributed equally to this work.

Figure S1. ^{1}H NMR (400 MHz, $CDCl_3$) spectrum of vermistatin ------------------------------------2

Figure S2. ^{13}C NMR (100 MHz, $CDCl_3$) spectrum of vermistatin ------------------------------------3

Figure S3. ^{1}H NMR (400 MHz, $CDCl_3$) spectrum of dihyrovermistatin ------------------------------3

Figure S4. ^{13}C NMR (100 MHz, $CDCl_3$) spectrum of dihyrovermistatin ----------------------------4

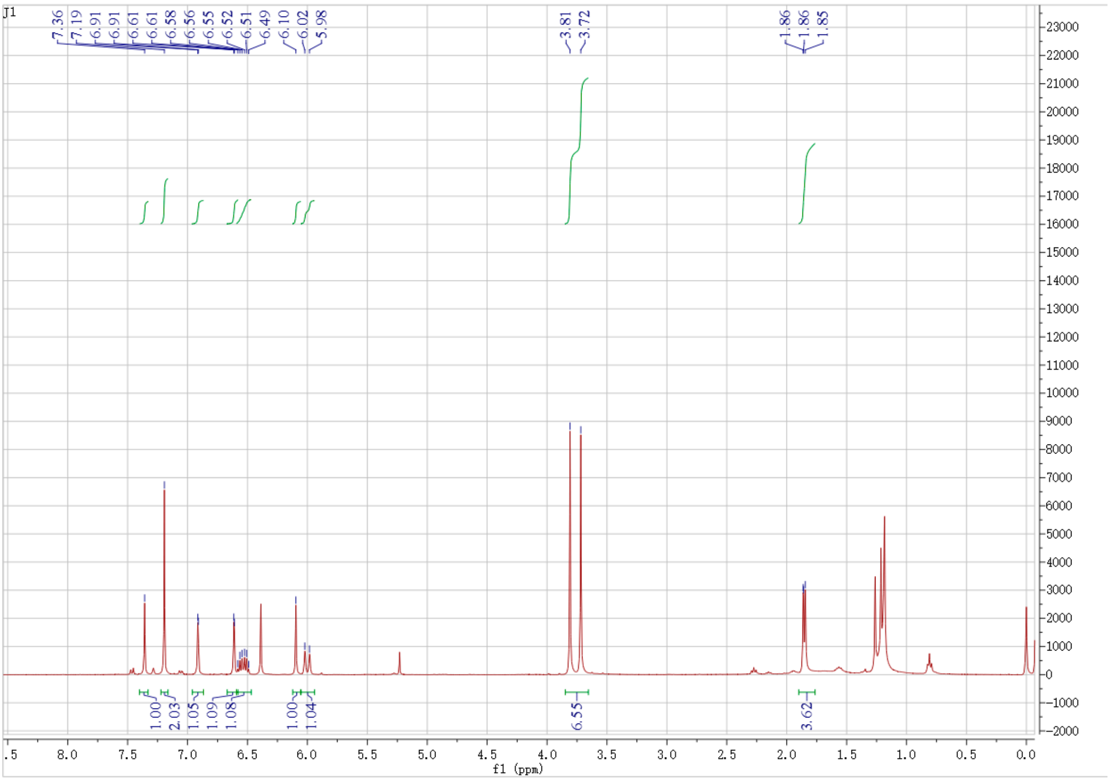

Figure S1. ^{1}H NMR (400 MHz, $CDCl_3$) spectrum of J1

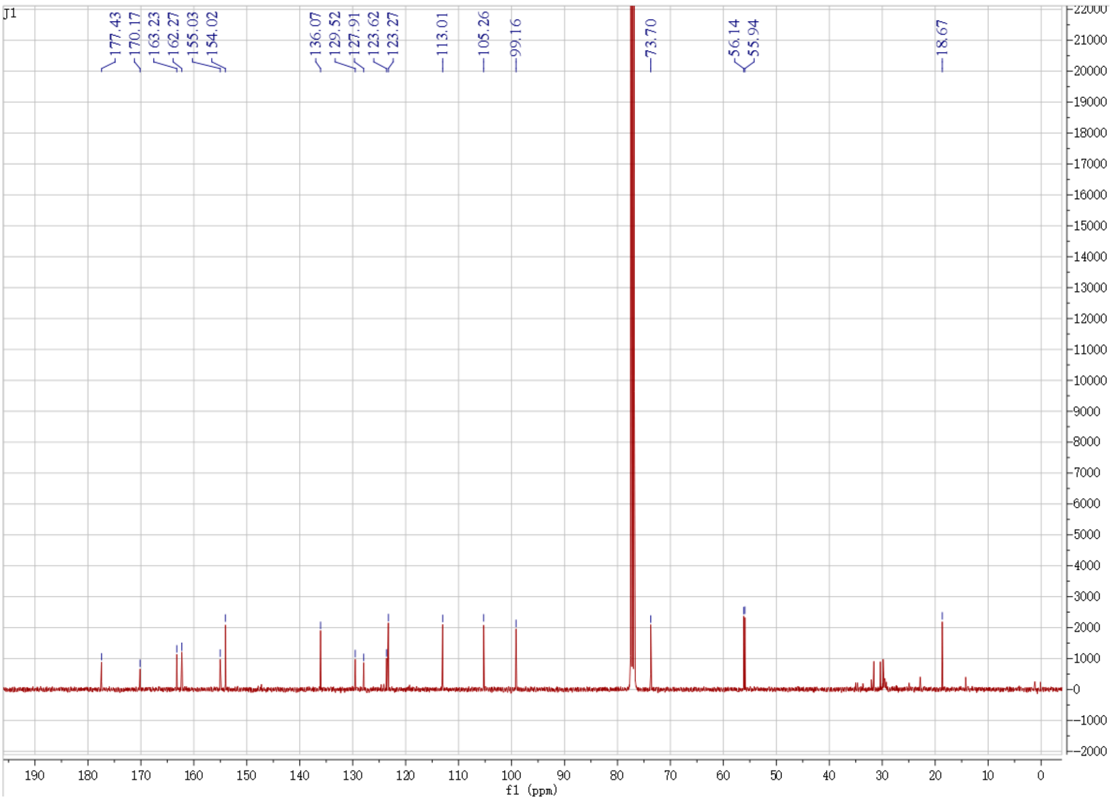

Figure S2. ^{13}C NMR (100 MHz, $CDCl_3$) spectrum of J1

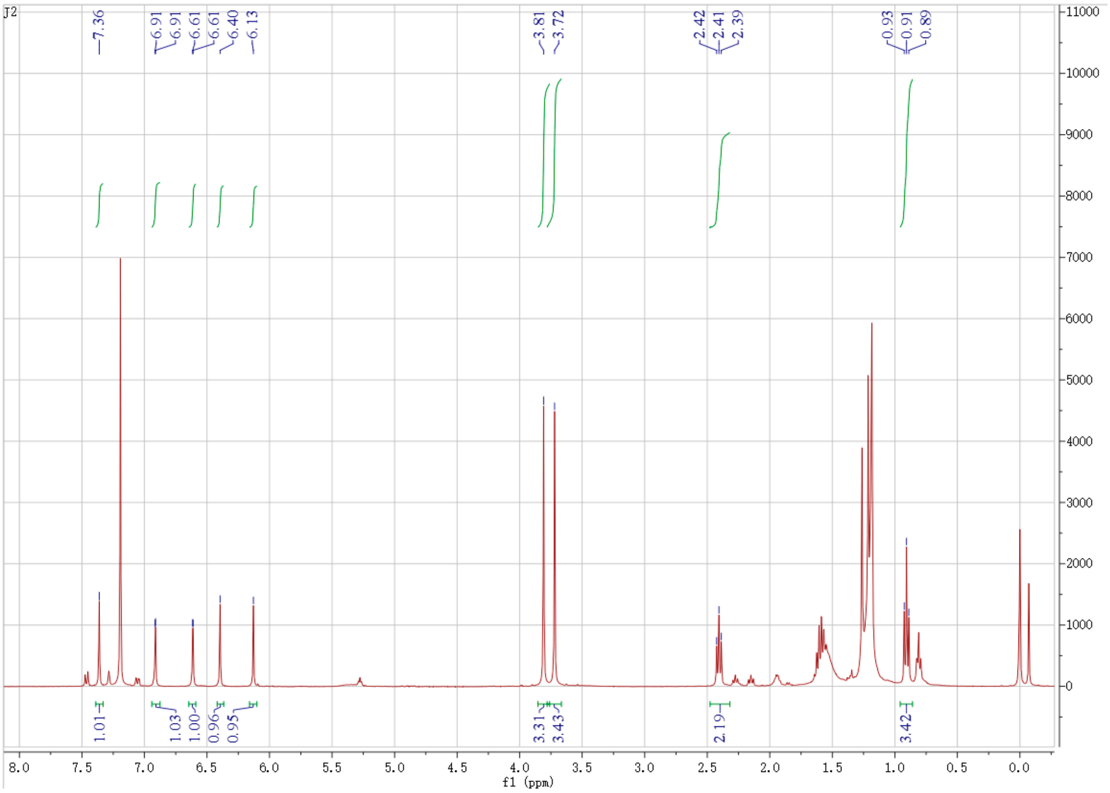

Figure S3. ^{1}H NMR (400 MHz, $CDCl_3$) spectrum of J2

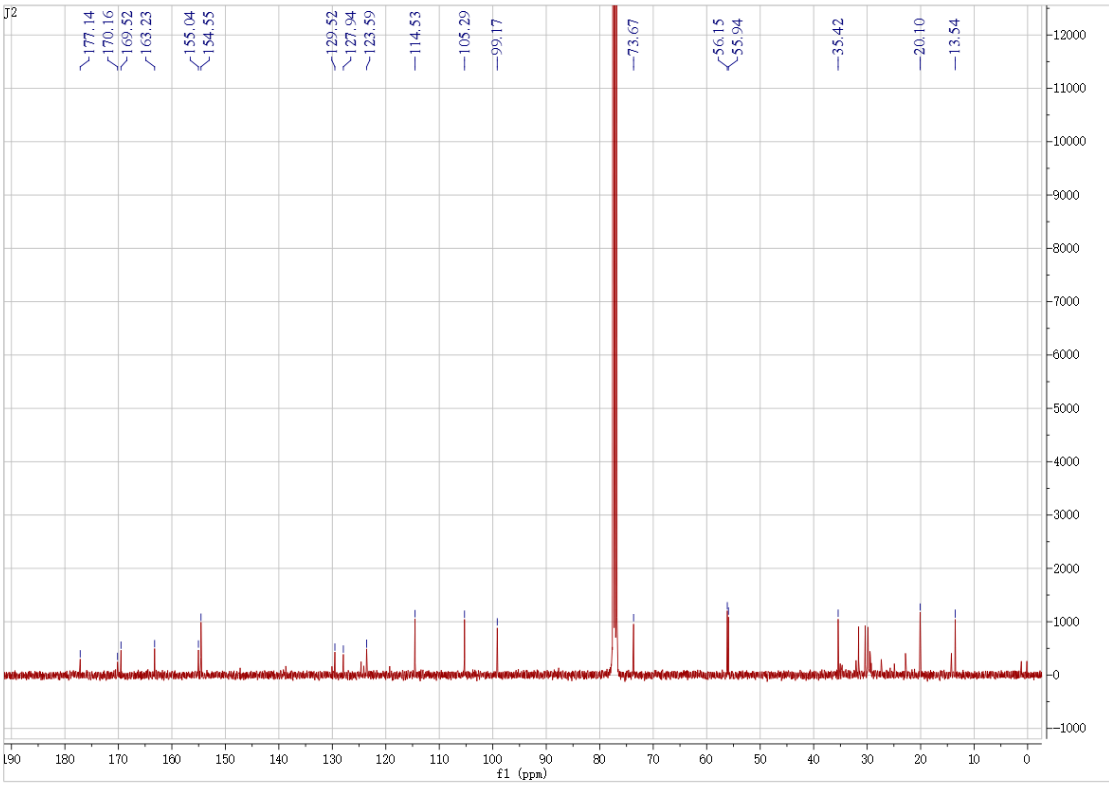

Figure S4. ^{13}C NMR (100 MHz, $CDCl_3$) spectrum of J2
